# Prognostic significance of anterior mitral valve leaflet length in individuals with a hypertrophic cardiomyopathy gene mutation without hypertrophic changes

**DOI:** 10.1007/s40477-018-0302-9

**Published:** 2018-06-06

**Authors:** Hannah G. van Velzen, Arend F. L. Schinkel, Myrthe E. Menting, Annemien E. van den Bosch, Michelle Michels

**Affiliations:** Department of Cardiology, Thorax-center, Erasmus Medical Center, ‘s-Gravendijkwal 230, 3015 CE Rotterdam, The Netherlands

**Keywords:** Echocardiography, Genetics, Hypertrophic cardiomyopathy, Mitral valve, Screening

## Abstract

**Purpose:**

Previous studies suggest that anterior mitral valve leaflet (AMVL) elongation is a primary phenotypic feature in hypertrophic cardiomyopathy (HCM). Our aim was to assess AMVL length in individuals with HCM gene mutations and in healthy controls and to identify predictors of the development of HCM during follow-up.

**Methods:**

A total of 133 HCM mutation carriers and 135 controls underwent cardiac examination including electro- and echocardiography. AMVL length was measured in the parasternal long axis and apical three chamber view during diastole. Univariate and multivariable cox proportional hazard regression analyses were performed to identify predictors of HCM.

**Results:**

There were no significant differences between HCM mutation carriers and controls regarding age and sex. In the parasternal long axis view, AMVL length was similar in mutation carriers and controls (24 ± 4 vs 24 ± 4 mm, *p* = 0.8). In the apical three chamber view, AMVL were shorter in mutation carriers (29 ± 4 vs 30 ± 4 mm, *p* = 0.02). When averaged for both views, AMVL length was similar in mutation carriers and controls (27 ± 3 vs 27 ± 3 mm, *p* = 0.2). During 5.8 ± 3.0 years follow-up, 13 (14%) HCM mutation carriers developed HCM. Pathological Q wave (hazard ratio 9.89, *p* = 0.004), E/e′ ratio (hazard ratio 2.52, *p* = 0.001), and maximal wall thickness (hazard ratio 2.15, *p* = 0.001) were independent predictors of HCM. AMVL length was not predictive of the development of HCM.

**Conclusions:**

AMVL length is similar in HCM mutation carriers and controls. AMVL length is not predictive of the development of HCM, in contrast to pathological Q wave, E/e′ ratio, and maximal wall thickness.

## SOMMARIO

**Obiettivi:**

Studi precedenti suggeriscono che l’allungamento della cuspide anteriore della valvola mitrale (LAVM) è una caratteristica fenotipica primaria nella cardiomiopatia ipertrofica (HCM). Il nostro obiettivo era quello di valutare la lunghezza del LAVM in individui con mutazioni del gene HCM e nei controlli sani e di identificare i predittori dello sviluppo di HCM durante il follow-up.

**Metodi:**

Un totale di 133 portatori di mutazione HCM e 135 controlli sono stati sottoposti ad un esame cardiologico che comprendeva un elettrocardiogramma ed ecocardiografia. La lunghezza del LAVM è stata misurata durante la fase diastolica in proiezione parasternale asse longitudinale ed in proiezione 3 camere apicali. Le analisi di regressione del rischio proporzionale coassiale univariata e multivariata sono state eseguite per identificare I predittori di HCM.

**Risultati:**

Non sono risultate differenze significative tra portatori di mutazione HCM e controlli per quanto riguarda età e sesso. Nella proiezione parasternale asse lungo, la lunghezza del LAVM era simile nei portatori di mutazione e controlli (24 ± 4 vs 24 ± 4 mm, *p* = 0.8). Nella proiezione 3 camere apicale, i valori del LAVM erano minori nei portatori di mutazione (29 ± 4 vs 30 ± 4 mm, *p* = 0.02). Eseguendo la media dei valori per entrambe le proiezioni, la lunghezza del LAVM era simile nei portatori di mutazione e nei controlli (27 ± 3 vs 27 ± 3 mm, *p* = 0.2). Durante un periodo di follow-up di 5.8 ± 3.0 anni, 13 (14%) dei portatori della mutazione HCM hanno sviluppato HCM. L’onda Q patologica (hazard ratio 9.89, *p* = 0,004), il rapporto E/e′ (hazard ratio 2.52, *p* = 0.001) e lo spessore massimale della parete (hazard ratio 2.15, *p* = 0.001) sono risultati predittori indipendenti di HCM. La lunghezza del LAVM non era predittiva di sviluppo di HCM.

**Conclusioni:**

La lunghezza del LAVM è simile nei portatori della mutazione HCM e nei controlli. La lunghezza del LAVM non è risultata predittiva dello sviluppo di HCM, a differenza dell’onda Q patologica, del rapporto E/e’, e dello spessore massimale della parete.

## Introduction

Hypertrophic cardiomyopathy (HCM) is a genetic cardiac disease with an estimated prevalence of 1:500–1:200 [1–3]. The diagnosis is based on the presence of a maximal wall thickness  ≥ 15 mm in index patients and  ≥ 13 mm in relatives, that is not solely explained by abnormal loading conditions [2]. A pathogenic HCM mutation is identified in 40–60% of patients with HCM [2, 4]. Presymptomatic genetic testing of relatives has led to the identification of HCM gene mutation carriers who do not fulfill the echocardiographic criterion of HCM [5]. HCM mutation carriers are at risk of developing HCM [5]. Conflicting data exists on whether the anterior mitral valve leaflets (AMVL) are elongated in mutation carriers and whether AMVL elongation is a predictor of the development of HCM during follow-up [6–11]. The aim of this study was to assess AMVL length in HCM mutation carriers and healthy controls and to determine the prognostic significance of AMVL length in HCM mutation carriers for the development of HCM during follow-up.

## Methods

### Study design and population

This single-center retrospective case–control and cohort study included 133 HCM mutation carriers without clinical expression of HCM who were clinically evaluated at our cardio-genetic outpatient clinic between the years 2004–2017. Genetic assessment and the family screening strategy at our center have been described previously [12, 13]. For comparison, 135 healthy controls underwent cardiac evaluation [14]. Controls were recruited via an advertisement. Inclusion criteria were normal physical examination, normal electrocardiography (ECG), and left ventricular (LV) ejection fraction  > 51%; exclusion criteria were prior cardiovascular disease or risk factors consisting of hypertension, diabetes mellitus, and hypercholesterolemia, systemic disease, medication known to influence cardiac function including thyroid medication (with the exception of asthma inhalers), professional athletes, body mass index  > 40 and women with breast implants [14]. The study conforms to the principles of the Declaration of Helsinki. All patients gave informed consent for inclusion in the registry and local institutional review board approval was obtained.

### Clinical evaluation

Clinical evaluation included medical history, physical examination, ECG, and transthoracic echocardiography. Standard 12-lead ECG was performed in the supine position during quiet respiration. LV hypertrophy was evaluated with the Romhilt–Estes criteria. Pathological Q waves were defined as duration  > 40 ms or depth  > 30% R wave in  ≥ 2 leads. T wave inversion was defined as  ≥ 3 mm in  ≥ 2 leads. Echocardiographic studies were analyzed according to the guidelines [15, 16]. Maximal wall thickness, left atrial size, leaflet and chordal systolic anterior motion of the mitral valve, and resting LV outflow tract peak velocity were assessed. LV outflow tract gradient was calculated with the Bernoulli equation. LV systolic function was categorized as: good (LV ejection fraction  > 51%), mildly reduced (LV ejection fraction 41–51%), moderately reduced (LV ejection fraction 30–40%), and poor (LV ejection fraction  < 30%) [16]. LV diastolic function was defined as normal, abnormal relaxation, pseudonormal or restrictive filling, based on Doppler mitral inflow pattern parameters including early (E) and late (A) LV filling velocities, E/A ratio, and tissue Doppler imaging-derived septal early diastolic velocities (e′) [17]. HCM during follow-up was defined as a maximal wall thickness ≥ 13 mm according to the guidelines [2].

### AMVL measurements

**Fig. 1:**
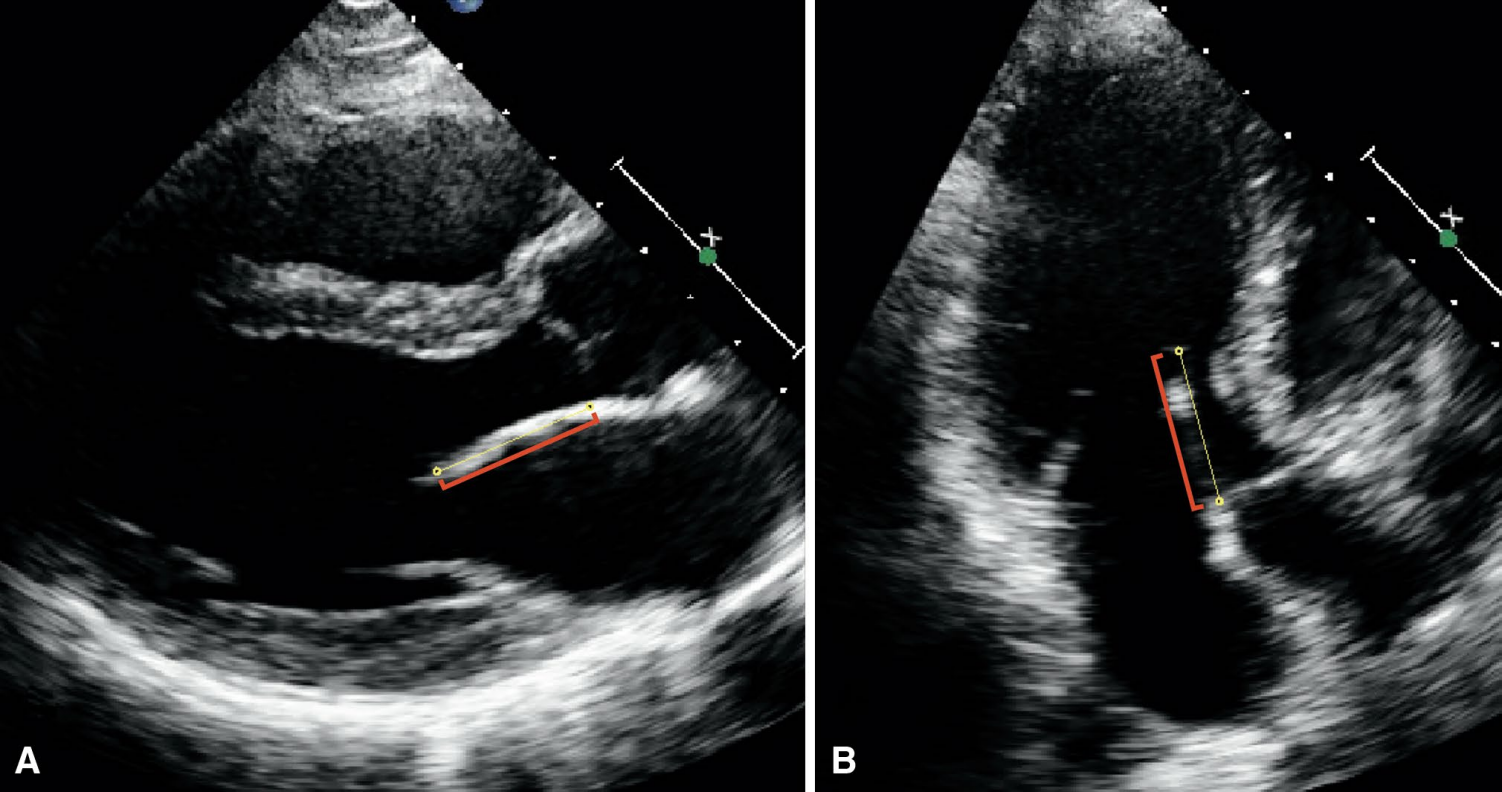
Example of anterior mitral valve leaflet length (AMVL) measurements in a hypertrophic cardiomyopathy gene mutation carrier without hypertrophic changes. In the parasternal long-axis view, **a** the AMVL measured 26 mm, and in the apical three chamber view, **b** the AMVL measured 26 mm

AMVL length was measured in the parasternal long axis (PLAX) view and in the apical three chamber (A3C) view, during diastole and with the leaflet maximally extended. In the PLAX view, leaflet length was defined as the distance from the tip of the leaflet to the junction between the anterior leaflet and the posterior aortic wall (hinge point), according to Klues et al. [18]. In the A3C view, leaflet length was defined as the distance from the tip of the leaflet to the insertion of the noncoronary aortic leaflet, according to Alhaj et al. [19]. Examples of AMVL measurements in both views are shown in Fig. 1. All AMVL measurements were performed by one reader. For intraobserver variability, one reader independently measured 40 AMVLs in the PLAX view and 40 AMVLs in the A3C view in an identical fashion on two occasions. For interobserver variability, two readers independently measured 20 AMVLs in the PLAX view and 20 AMVLs in the A3C view.

### Statistical methods

The statistical analysis was performed using SPSS 21 (IBM, Armonk, NY). Normally distributed continuous data are expressed as mean ± standard deviation and non-normally distributed data as median followed by interquartile range. For comparing categorical variables Pearson’s Chi-square test was used. For comparing continuous variables *t* test was used, and Mann–Whitney U in case of non-normally distributed data. All analyses were two-sided; *p* values  < 0.05 were considered significant. Inter-observer and intra-observer agreement was defined as the mean of the difference between two measurements ± standard deviation. Univariate and multivariable cox proportional hazard regression was performed to determine hazard ratios (HR) and 95% confidence intervals (CI). After screening for multicollinearity, the univariate significant variables with the highest HR were entered into the multivariable regression model. To calculate the allowed number of variables for inclusion in the multivariable analysis, the square root of the number of events was used. This is an alternative method to determine the number of variables allowed for inclusion in the multivariable analysis [20].

## Results

### Clinical evaluation

HCM gene mutation carriers represented mutations in 10 different genes. The *MYBPC3* gene was most frequently affected (77%), followed by the *MYH7* gene (11%). Other genes affected were *TNNT2* (3%), *MYL2* (2%), *FHL1* (2%), *ALPK3* (2%) *MIB1* (0.75%), *TNNI3* (0.75%), *TPM1* (0.75%), and *MYL3* (0.75%). Clinical and echocardiographic characteristics in mutation carriers and controls are presented in Table 1. Mutation carriers and controls had similar age, gender, and body surface area. Compared to controls, more mutation carriers had pathological Q waves (4 vs 0%, *p* = 0.02), and mutation carriers had a higher E/e′ ratio (8.2 ± 1.9 vs 7.7 ± 1.9, *p* = 0.03), and a higher maximal wall thickness (8.9 ± 1.9 vs 8.0 ± 1.8 mm, *p* = 0.001).

**Table 1:** Clinical and echocardiographic characteristics of the study population

Variable	Mutation carrier (n = 133)	Control (n = 135)	*p* value
Age (year)	41 ± 14	44 ± 14	0.11
Female gender *n* (%)	85 (64)	73 (54)	0.18
Body surface area (m^2^)	1.9 ± 0.2	1.9 ± 0.2	0.86
Electrocardiography			
Romhilt–Estes ≥ 4 *n* (%)	10 (8)	4 (3)	0.09
T wave inversion *n* (%)	1 (1)	0 (0)	0.31
Pathological Q wave *n* (%)	5 (4)	0 (0)	0.02
Echocardiography			
Maximal wall thickness (mm)	8.9 ± 2.0	8.0 ± 1.8	<0.001
Left atrial size (mm)	34 ± 5	34 ± 4	0.24
LVOT gradient ≥ 30 mmHg^a^ *n* (%)	0 (0)	0 (0)	0.50
AMVL, PLAX (mm)	24 ± 4	24 ± 4	0.85
AMVL, A3C (mm)	29 ± 4	30 ± 4	0.02
AMVL, averaged (mm)	27 ± 3	27 ± 3	0.17
Chordal systolic anterior motion *n* (%)	5 (4)	1 (1)	0.09
Leaflet systolic anterior motion *n* (%)	0 (0)	0 (0)	0.50
E/A ratio	1.4 ± 0.5	1.6 ± 0.7	0.03
E/e’ ratio	8.2 ± 1.9	7.7 ± 1.9	0.03
Septal e’ (cm/s)	9.5 ± 2.5	9.6 ± 2.6	0.61
Diastolic function			
Normal *n* (%)	105 (83)	111 (85)	0.66
Abnormal relaxation *n* (%)	10 (8)	7 (5)	0.39
Pseudonormal filling *n* (%)	11 (9)	13 (10)	0.76
Restrictive filling *n* (%)	0 (0)	0 (0)	0.50
Systolic function			
Good *n* (%)	132 (99)	135 (100)	0.31
Mildly reduced *n* (%)	1 (1)	0 (0)	0.31
Moderately reduced *n* (%)	0 (0)	0 (0)	0.50
Poor *n* (%)	0 (0)	0 (0)	0.50

Data are expressed as mean ± standard deviation or as absolute and (%)

*AMVL* anterior mitral valve leaflet, *A3C* apical three chamber view, *LVOT* left ventricular outflow tract, *PLAX* parasternal long-axis view

^a^at rest

### AMVL measurements

**Fig. 2:**
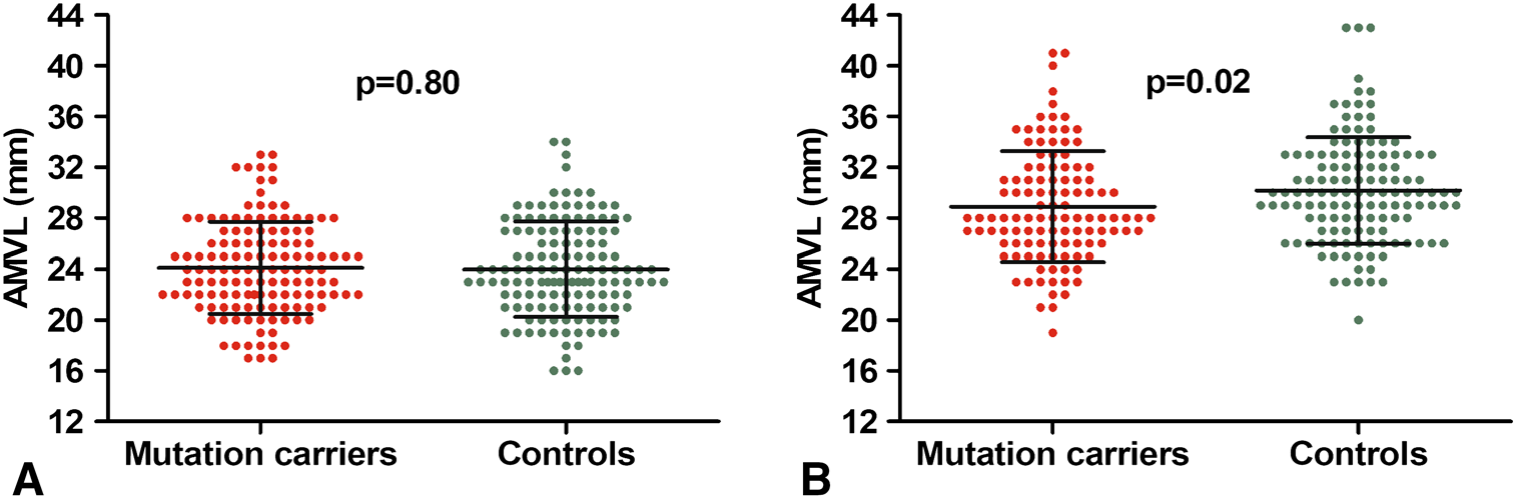
Beeswarm plot of anterior mitral valve leaflet (AMVL) length measurements in hypertrophic cardiomyopathy gene mutation carriers without hypertrophic changes versus healthy controls, assessed with transthoracic echocardiography in the **a** parasternal long-axis view and **b** apical three chamber view

Beeswarm plots of AMVL measurements in the PLAX and the A3C view are presented in Fig. 2. In the PLAX view, AMVL length did not differ between mutation carriers and controls (24 ± 4 vs 24 ± 4 mm, *p* = 0.8). In the A3C view, AMVL were shorter in the mutation carriers (29 ± 4 vs 30 ± 4 mm, *p* = 0.02). When averaged for both views, AMVL length was similar in mutation carriers and controls (27 ± 3 vs 27 ± 3 mm, *p* = 0.2). Overall, AMVL were significantly longer in the A3C view than in the PLAX view (30 ± 4 vs 24 ± 4 mm, *p* < 0.001).

### Intra-observer and inter-observer agreement

In the PLAX view, the inter-observer agreement was − 2.7 ± 2.6 mm and the intra-observer agreement was − 1.0 ± 3.5 mm. In the A3C view, the inter-observer agreement was 2.0 ± 2.5 mm and the intra-observer agreement was 0.5 ± 2.6 mm.

### Follow-up

During 5.8 ± 3.0 years follow-up, 13 (14%) mutation carriers developed HCM. Mean age at HCM diagnosis was 52 ± 17 years. In these 13 mutation carriers, maximal wall thickness increased from a median 10 (interquartile range 8, 11) mm to 13 (interquartile range 13, 14) mm, with a mean rate of 0.7 ± 0.3 mm/year. Table 2 presents the baseline characteristics in those who developed HCM during follow-up and those who did not. Univariate significant predictors of the development of HCM were pathological Q wave (HR 9.74, 95% CI 2.53–37.46, *p* = 0.001), maximal wall thickness (HR 1.64, 95% CI 1.12–2.39, *p* = 0.01), E/e′ ratio (HR 1.63, 95% CI 1.20–2.23, *p* = 0.002), left atrial size (HR 1.16, 95% CI 1.03–1.31, *p* = 0.01), and age (HR 1.06, 95% CI 1.02–1.11, *p* = 0.01). AMVL length was not predictive of the development of HCM in the PLAX view (HR 1.03, 95% CI 0.87–1.22, *p* = 0.72) or in the A3C view (HR 0.99, 95% CI 0.86–1.15, *p* = 0.92). Multivariable cox regression analysis which included three variables demonstrates that pathological Q wave (adjusted HR 9.89, 95% CI 2.09–46.95, *p* = 0.004), E/e′ ratio (adjusted HR 2.52, 95% CI 1.48–4.29, *p* = 0.001), and maximal wall thickness (adjusted HR 2.15, 95% CI 1.36–3.42, *p* = 0.001) all were independent predictors of HCM during follow-up.

**Table 2:** Baseline characteristics of HCM gene mutation carriers who did and did not develop hypertrophic cardiomyopathy during follow-up

Variable	Developed HCM		
	Yes (*n* = 13)	No (*n* = 77)	*p* value
Age (years)	47 ± 19	39 ± 13	0.05
Male gender *n* (%)	8 (62)	25 (33)	0.04
Romhilt–Estes ≥ 4 *n* (%)	1 (8)	5 (7)	0.87
T wave inversion *n* (%)	1 (8)	0 (0)	0.01
Pathological Q *n* (%)	3 (23)	2 (3)	0.003
Left atrial size (mm)	39 ± 5	33 ± 5	<0.001
Maximal wall thickness (mm)	10 ± 2	9 ± 2	0.02
AMVL, PLAX (mm)	25 ± 5	24 ± 3	0.47
AMVL, A3C (mm)	29 ± 5	29 ± 4	0.90
Chordal systolic anterior motion *n* (%)	1 (8)	3 (4)	0.54
E/e’ ratio	9.3 ± 1.8	8.1 ± 1.7	0.03
Septal e’ (cm/s)	8.5 ± 2.7	9.5 ± 2.6	0.20
Abnormal diastolic function *n* (%)	4 (31)	11 (16)	0.21

Data are expressed as mean ± standard deviation or as absolute and (%)

*AMVL* anterior mitral valve leaflet, *A3C* apical three chamber view, *HCM* hypertrophic cardiomyopathy, *PLAX* parasternal long axis view

## Discussion

During HCM family screening, individuals who carry a HCM gene mutation may not fulfil the echocardiographic diagnostic criterion of HCM [5]. Because of the age-related penetrance of HCM, long-term clinical follow-up including ECG and echocardiography is recommended [2, 3]. Currently, it is unclear which HCM mutation carriers will develop HCM [2, 11]. We aimed to assess AMVL length in mutation carriers and controls, and determine the prognostic value of AMVL length for the development of HCM during follow-up. Our main findings are: (1) AMVL length is similar in mutation carriers and controls, (2) AMVL length is not predictive of the development of HCM, and (3) pathological Q wave, E/e′ ratio, and maximal wall thickness are independent predictors of the development of HCM.

### AMVL elongation is not a primary phenotypic feature of HCM

In patients with HCM, AMVL elongation has been demonstrated pathologically, on echocardiography, and on cardiovascular magnetic resonance imaging [10, 18, 21–23]. Among other factors, AMVL elongation contributes to systolic anterior motion of the mitral valve and LV outflow tract obstruction [23–25]. The etiology of AMVL elongation in patients with HCM is unclear. Both the pathological study of Klues et al. and the in vivo study of Kim et al. found that AMVL elongation is not secondary to LV outflow tract obstruction or systolic anterior motion of the mitral valve, since it also occurs in patients without LV outflow tract obstruction or systolic anterior motion [19, 21, 22]. Therefore, it was suggested that AMVL elongation is a primary phenotypic expression of HCM. Several studies have indeed reported AMVL elongation in mutation carriers as measured by magnetic resonance imaging [6, 7, 10], and echocardiography [9]. The current study contradicts these findings. In line with our findings, a recent magnetic resonance imaging study similarly reported no difference in AMVL length between mutation carriers and controls [8]. The discrepancy between the studies may be related to the small number of participants, different imaging modalities used, different distribution of genetic mutations, or different methodologies used for AMVL measurements.

For several reasons, we believe it is unlikely that HCM gene mutations cause AMVL elongation. First, there are no sarcomeric proteins in the mitral valve leaflet [25]. Second, a HCM animal model including heterozygous cardiac myosin-binding protein C targeted knock-out mice embryos did not show mitral leaflet elongation [26]. Third, Captur et al. observed AMVL elongation in genotype-negative patients with HCM [10]. And finally, most morphological studies demonstrate that mitral leaflets are intrinsically normal [21, 27]. Other potential etiologies of AMVL elongation are being investigated, such as the paracrine effects from the abnormal LV wall which influences valvulogenesis, or the abnormal differentiation of pluripotent epicardial-derived cells into fibroblast-cells with increased synthesis of periostin which might drive leaflet elongation [25].

### Predicting the development of HCM

The current study demonstrates that AMVL length had no predictive value for the development of HCM. Hence, AMVL length cannot be used as a preclinical marker of the development of HCM. Similar observations were made in a prior smaller study by Ho et al. [28]. Pathological Q wave had a high predictive value for the development of HCM. Indeed, prior investigation of ECGs in genotyped HCM populations demonstrated that Q waves and repolarization abnormalities are the most distinguishing ECG manifestations of sarcomere mutations [29]. However, the clinical utility of Q waves is probably limited because of the low negative predictive value; 10 out of 13 mutations that developed HCM did not have pathological Q waves at baseline. Our study did not demonstrate a prognostic value of septal e′, in contrast to Ho et al. [28]. The age difference between the studies (16 vs 41 years) and differences in genetic mutations might explain this discrepancy. However, we did observe a predictive value of E/e′ ratio, which supports the suggestion that diastolic dysfunction is a primary phenotypic feature of HCM [28, 30].

### Technical challenges associated with AMVL measurement by echocardiography

Previous studies most commonly used magnetic resonance imaging to measure AMVL length [6–8, 10]. Since transthoracic echocardiography is the advised imaging modality in HCM clinical screening strategies and has a higher spatial and temporal resolution than cardiac magnetic resonance imaging [31, 32], we used echocardiography to determine AMVL length. Overall, inter-observer variability in both views was 2–3 mm, similar to previous studies [6, 8, 18]. The difference between observers may be explained by the technical difficulty of distinguishing the mitral leaflet from the chordae tendineae, and by the frame-to-frame variability in AMVL length caused by AMVL movement during diastole and respiration. Intra-observer agreement was best for the A3C view, which was unexpected because in the PLAX view the distance to the transducer is shorter. It may be explained by the landmarks that were used; in the A3C view the insertion of the noncoronary aortic leaflet is more easily identifiable in comparison to the hinge point in the PLAX view. Finally, AMVL were significantly longer in the A3C view than in the PLAX view; the measurement in the A3C view included the intervalvular fibrosa.

### Limitations

This study has several limitations. First, although the study population is large compared to previous studies, a higher sample size would reduce the risk of sampling error. Second, the proportion of HCM gene mutation carriers that developed HCM during follow-up was limited.

## Conclusions

AMVL length is similar in HCM mutation carriers and healthy controls. AMVL length is not a predictor of the development of HCM during follow-up, in contrast to pathological Q wave, E/e′ ratio, and maximal wall thickness.
